# Extrathoracic chronic heamatoma presenting as a chest wall tumor 2 years after a blunt thoracic injury

**DOI:** 10.1186/1749-8090-6-156

**Published:** 2011-11-23

**Authors:** Vasileios K Kouritas, Antonios G Roussakis, Konstantinos Soultanis, Ion Bellenis

**Affiliations:** 1 Department of Thoracic and Vascular Surgery, 'Evangelismos' Hospital, Athens, Greece

**Keywords:** Chronic heamatoma, extrathoracic, soft tissue tumor, giant

## Abstract

Chronic expanding heamatomas may present as masses mimicking chest wall tumors. We report the case of a patient who was presented with a giant posterior extrathoracic chest wall tumor. The mass was proven to be a chronic heamatoma possibly developed after a blunt thoracic injury which took place 2 years before presentation and was growing thereafter. Clinicians should have high suspicion of rare entities which mimic tumors and consider any information reported by the patient's history in their diagnostic process.

## Background

Uncommon entities are rarely presented as lesions or masses, mimicking thoracic or pleural tumors, leading clinicians to establish wrong diagnosis [1-3].

Hemorrhagic manifestations complicate falls, which occur more frequently in older patients, who receive anticoagulant therapy for different pathologies. The commonest presentation of such hemorrhagic manifestations is cuts and bruising, with more serious complications, such as intracranial heamatomas or bleeding because of fractures being also not uncommon among such patients [4]. Thoracic manifestations, such as the formation of chest wall heamatomas are extremely rare [2,4].

We herein report a case of a male patient who was presented with a giant posterior chest wall tumor, which was eventually proven to be a growing chronic heamatoma due to a blunt thoracic injury 2 years before presentation.

## Case Presentation

**Figure 1:**
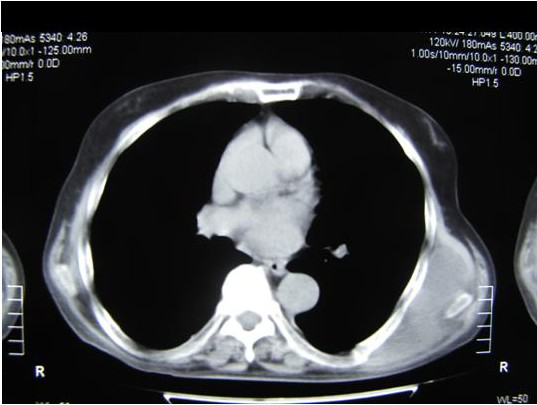
**Computed Tomography of the patient showing the extrathoracic mass on the left posterior thoracic cage**.

A 76 year old male was presented to our department with a huge mass (20 × 15 cm) in his left posterior thoracic cage, extending between the medial edge of the scapula to the spine. The patient reported that the mass appeared 2 years ago and that it was slowly growing bigger ever since. From his past medical history, he had a myocardial infarction treated with angioplasty for which he was on a regime of acetyl-salicylic acid, with a dosage of 325 mg once per day. He also reported an ipsilateral chest wall injury after a fall 2 years ago for which he did not seek medical help. On examination the mass's texture was solid and fixed on the chest wall, inflicting pain to the patient who additionally complained of cosmetic discomfort. The patient was subjected to a thoracic computed tomography which revealed a huge extrathoracic tumor extending (medially) from the left side of the spine (laterally) to the tip of the scapula and (superiorly) below the scapula to the anterolateral thoracic wall (inferiorly). The mass did not seem to enter the thoracic cage or to invade the ribs and it had a tissue density (Housfield Units 40-50) of fat and muscle (Figure 1). Based on a) the CT findings raising the suspicion of malignancy and ensuring total excision of the mass, b) the preference of the patient who did not consent to major surgery and c) the presenting symptoms (pain, cosmetic), a surgical removal - open biopsy of the lesion was decided.

**Figure 2:**
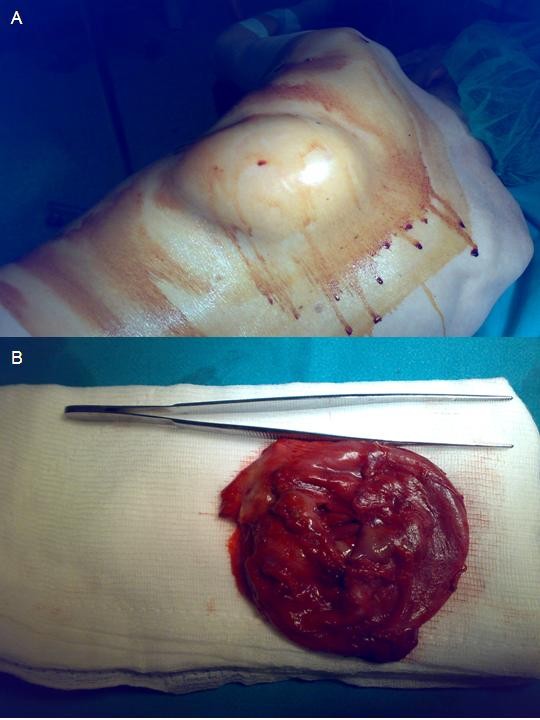
**Intra-operative images of the patient who is positioned with his left hemithorax on top (A) and of the excised specimen after evacuation of its contents (B)**.

Intra-operatively, the mass (Figure 2A) was dissected off the chest wall below the latissimus dorsi and trapezius muscles and was resected en bloc. Two broken ribs were palpated at the site where the mass was firmly attached to the thoracic cage. A drain was left in site. After the completion of the operation, the mass was opened and blood (fresh and organized) exited with the presence of a few clots (Figure 2B).

The patient's recovery was uneventful. The drain was removed the day after the operation and the patient was discharged two days after the operation.

The mass was proven to be a heamatoma, as per the histopathology report with the mass being a calcified fibrous capsule without neoplasia.

## Conclusions

Different pathologies of the thorax, rarely, may be presented as chest wall tumors. Chest wall heamatomas should be considered in elder patients on anticoagulation therapy sustaining trauma.

Heamatomas may rarely be organized to mimic soft tissue tumors [1-3]. This is the case, especially when the heamatoma is chronic or expanding, without developing the clinical symptoms and signs of an acute heamatoma such as collapsus and hypovolunemia [1-3]. The size of such heamatomas may obscure the diagnosis; they can grow up to giant masses that can eventually occupy the whole hemithorax leading to hypoventilation and respiratory failure, mimicking in this way large sarcomas. Additionally, heamatomas may be calcified, event that may produce a malignant appearance, especially at imaging and eventually confuse the correct diagnosis [3]. In our case the heamatoma had all the above characteristics of a soft tissue tumor and therefore the pre-operative diagnosis was misled.

Almost all cases reported in the literature involve heamatomas developing within the pleural cavity [2,3]. In our case the heamatoma was developed outside the thoracic cage, below the muscles, giving the appearance of an expanding tumor.

Chronic expanding heamatomas usually develop over a clinical background, such as tuberculosis, with patients forming an appearance of a heamatoma, many years after the tuberculus manifestation [3,5,6]. In most of these cases the patients are reported to be presented in a critical condition requiring emergency surgery and excision of the expanding heamatoma. Surprisingly, the onset of our patient's heamatoma expansion was gradual and consequently it was organized.

Patients receiving anticoagulation treatment are theoretically under an increased danger of bleeding and therefore developing heamatomas [4]. The most common presentation of hemorrhagic complications is major cuts needing intervention, or extensive bruising. Intracranial hemorrhage and hemorrhage following fractures are also usually diagnosed in anticoagulant patients who have sustained trauma [4]. However, hemorrhagic complications in patients receiving anticoagulation therapy may involve rare sites, which are usually automatically inflicted [7]. Thoracic complications in anticoagulation treatment patients are rare and usually regard to extra-pleural heamatomas [8] or development of a hemothorax. In our case the patient had a simple thoracic injury after a fall, which gradually evolved in a large extra-thoracic heamatoma, without however presenting symptoms and signs i.e. a bruise.

The type of anticoagulation treatment is reported to be important for the hemorrhagic phenomena, with warfarin being the drug inflicting the most severe hemorrhagic complications [4,9]. Although this notion is recently questioned, it seems that other anticoagulants, such as the acetyl-salicylic acid and clopidogrel, may also inflict major bleeding complications in patients sustaining trauma [4,10]. This knowledge may explain the appearance of the chest wall heamatoma in our patient who sustained a fall.

In conclusion, heamatomas may mimic masses, a fact that clinicians should keep in mind, and therefore should consider all history and examination data in order to establish a diagnosis before proceeding to surgery.

## Consent

The patient has given written consent for his clinical and imaging details to be published in this Case Report. The signed consent is at the disposal of the Editor for inspection.

## Competing interests

The authors declare that they have no competing interests.

## Authors' contributions

VKK, AGR, KS and IB operated the patient. VKK wrote the case report. IB edited the text. All authors read and approved the revised manuscript.
